# Complete Genome Sequence of Salmonella enterica subsp. enterica Serotype Derby, Associated with the Pork Sector in France

**DOI:** 10.1128/MRA.01027-18

**Published:** 2018-09-27

**Authors:** Yann Sévellec, Sophie A. Granier, Nicolas Radomski, Arnaud Felten, Simon Le Hello, Carole Feurer, Michel-Yves Mistou, Sabrina Cadel-Six

**Affiliations:** a Université Paris-Est, Marne-la-Vallée, France; b Institut Pasteur, Centre National de Référence des *Salmonella*, Unité des Bactéries Pathogènes Entériques, Paris, France; c French Institute for the Pig and Pork Industry (IFIP), Le Rheu, France; d ANSES, Laboratory for Food Safety, Maisons-Alfort, France; Iowa State University

## Abstract

In the European Union, Salmonella enterica subsp. enterica serovar Derby is the most abundant serotype isolated from pork. Recent studies have shown that this serotype is polyphyletic. However, one main genomic lineage, characterized by sequence type 40 (ST40), the presence of the Salmonella pathogenicity island 23, and showing resistance to streptomycin, sulphonamides, and tetracycline (STR-SSS-TET), is pork associated. Here, we describe the complete genome sequence of a strain from this lineage isolated in France.

## Abstract (precis)

In the European Union, Salmonella enterica subsp. enterica serovar Derby is the most abundant serotype isolated from pork.

## ANNOUNCEMENT

In the European Union, Salmonella enterica subsp. enterica serovar Derby (*S.* Derby) was the fifth serovar reported from human cases of salmonellosis in 2016 (0.7%; 325/44,462 confirmed cases) (1). European monitoring data linked this serovar predominantly to pigs and pork meat and, to a lesser extent, turkey and cattle (1). Recent studies based on whole-genome sequencing (WGS) have shown that distinct genomic lineages of *S.* Derby exist, associated with either pork or poultry (2, 3). The main genomic lineage associated with pork is characterized by multilocus sequence typing (MLST) profile 40 (sequence type 40 [ST40]), presence of genes mediating resistance to aminoglycosides, sulfonamides, and tetracyclines (3), and presence of *Salmonella* pathogenicity island 23 (SPI-23), which was previously associated with pork enterocyte invasion (2). We present here the complete genome sequence of S. enterica subsp. enterica serovar Derby strain 2014LSAL02547, which represents this genomic lineage.

Strain 2014LSAL02547 was isolated in 2014 from a pig carcass sampled at a slaughterhouse in Brittany, France, and identified as belonging to *Salmonella* serovar Derby, according to the White-Kauffmann-Le Minor scheme (4). Its genome was sequenced using Illumina HiSeq (i.e., paired-end read sequencing, 2 × 150 bp) and PacBio (i.e., long-read sequencing) technologies. Concerning the Illumina HiSeq sequencing, genomic DNA was isolated from overnight culture at 37°C on a tryptone soy yeast extract agar plate using the Wizard genomic DNA purification kit (Promega, France) according to the manufacturer’s instructions for Gram-negative organisms. The DNA concentration was measured with a Qubit fluorometer, and a gel of 0.8% agarose was used to assess the quality of the extraction (and an eventual degradation of the DNA). Library preparation and sequencing were performed by the Institut du Cerveau et de la Moelle épinière (www.icm-institute.org) using NextEra XT technology and a NextSeq 500 sequencer, respectively (Illumina). Concerning the PacBio sequencing, DNA extraction and library preparation were performed by Genoscreen (Lille, France). DNA was extracted using a Gentra Puregene kit (Qiagen), the DNA concentration was assessed using a Qubit fluorometer, and the DNA extract’s quality was checked by agarose gel electrophoresis. Library preparation was made by DNA fragmentation and ligation of single-molecule real-time (SMRT) adaptors. Prior to sequencing, BluePippin size selection (Sage Science) was set at 15 kb in order to achieve identical sequence overlaps. PacBio sequencing was performed on one SMRT cell. Quality of the Illumina and PacBio reads was examined using FastQC v0.11.5 (5). Prinseq v0.20.4 (6) was used to select Illumina long reads of good quality (no undefined bases; Phred, >30; length, >60 kb). SMRT Analysis v2.3.0 software was used to assemble PacBio reads. In SMRT Analysis, the Hierarchical Genome Assembly Process (HGAP) v3.0 (7) was invoked to correct the subreads (length, >1,000 bases; read score, 0.8), and Celera v8.3 (8) was used for assembly (subread length, >500 bases; deep coverage, >25×). SAMtools v1.5 (9) was used to map the Illumina short paired-end reads against the PacBio assembly to correct potential assembly mistakes and to determine the depth of the final assembly. The final deep coverage obtained was 146×. A unique 4.86-Mb contig was obtained, with a GC content of 51.12%. The genome was annotated using Prokka (10). It included 4,549 coding sequences (CDS) and 88 tRNAs.

Genes mediating antimicrobial resistance phenotype STR-SSS-TET (showing resistance to streptomycin, sulfonamides, and tetracycline) are part of the *Salmonella* genomic island 1 (SGI-1) (3), described in Table 1. This SGI-1 element integrated a cluster of mercury resistance genes (*merA*, *merC*, *merP*, *merT*, and *merR*) located in a Tn*7* transposon. SPI-23 from the 2014LSAL02547 genome was 36,603 bp long and was located between the *DAD50_12070* and *mftA* genes (Table 1).

**TABLE 1:** Structure of the SGI-1C and SPI-23 from *S.* Derby strain 2014LSAL02547^a^

Genomic island and position	CDS	Protein	Length (bp)
SGI-1C			
1	*int*	Integrase	1,158
2	s002	Helix-turn-helix domain protein	327
3	*rep*	Replication protein	954
4	S004	Hypothetical protein	228
5	S005	Hypothetical protein	2,760
6	S006	Hypothetical protein	534
7	S007	Hypothetical protein	612
8	S008	Hypothetical protein	210
9	S009	Hypothetical protein	291
10	S010	Hypothetical protein	255
11	*traG*	Pilus assembly protein TraG	3,405
12	S012	Conjugative relaxosome accessory transposon protein	1,425
13	S013	Hypothetical protein	858
14	S014	Hypothetical protein	411
15	S015	Hypothetical protein	270
16	S016	Hypothetical protein	213
17	S017	Hypothetical protein	243
18	*intI1*	Phage integrase	966
19	prokka_3715	Hypothetical protein	645
20	*aplIR*	Type 2 restriction enzyme AplI	1,092
21	*taqIM*	Modification methylase TaqI	1,830
22	*hin*	DNA-invertase hin	588
23	*intI1*	Integrase Int1	1,014
24	*aadA2*	Streptomycin 3″-adenylyltransferase	780
25	*qacEdelta1*	Quaternary ammonium compound efflux small multidrug resistance transporter QacE delta 1	348
26	*sul1*	Sulfonamide-resistant dihydropteroate synthase	840
27	*ypeA*	Putative acetyltransferase	501
28	*intB*	Transposase/IS protein	786
29	*istA*	Integrase core domain IS*26*	1,515
30	*tniB*	Bacterial TniB protein	861
31	*tnsB*	Transposon Tn*7* transposition protein TnsB	1,680
32	*cph2*	Phytochrome-like protein cph2	708
33	*merE*	Mercury resistance protein MerE	237
34	*mta*	Zinc-responsive transcriptional regulator	363
35	*merA*	Mercuric reductase	1,695
36	*merC*	Mercuric resistance protein MerC	423
37	*merP*	Mercuric transport protein periplasmic component precursor	276
38	*merT*	MerT mercuric transport protein	351
39	*merR*	Mercuric resistance operon regulatory protein	435
40	prokka_3736	Hypothetical protein (putative relaxase)	243
41	*tetR*	Tetracycline repressor protein class A from transposon 1721	678
42	*tetA*	Tetracycline resistance protein, class C	1,200
43	*yedA*	Putative inner membrane transporter YedA	783
44	*tnsB*	Transposon Tn*7* transposition protein TnsB	708
45	prokka_3741	Hypothetical protein	1,035
SPI-23			
1	*intA3*	Integrase A	1,275
2	prokka_01810	Hypothetical protein	252
3	prokka_01811	Abortive infection phage resistance protein	1,113
4	prokka_01812	Bacterial shuffle protein	1,488
5	prokka_01813	Major subunit of bundle-forming pilus precursor	558
6	*bfpA*	Conjugal transfer protein TraD	306
7	*traD*	Hypothetical protein	1,515
8	prokka_01816	Hypothetical protein	735
9	prokka_01817	Hypothetical protein	576
10	prokka_01818	Hypothetical protein	531
11	prokka_01819	Hypothetical protein	342
12	prokka_01820	Hypothetical protein	471
13	prokka_01821	Hypothetical protein	552
14	prokka_01822	Hypothetical protein	180
15	prokka_01823	Hypothetical protein	240
16	prokka_01824	Hypothetical protein	1,251
17	prokka_01825	Hypothetical protein	282
18	prokka_01826	Hypothetical protein	438
19	prokka_01827	Hypothetical protein	558
20	prokka_01828	Hypothetical protein	183
21	prokka_01829	Hypothetical protein	477
22	prokka_01830	Hypothetical protein	2,730
23	prokka_01831	Hypothetical protein	504
24	prokka_01832	Hypothetical protein	93
25	prokka_01833	Hypothetical protein	870
26	prokka_01834	Hypothetical protein	588
27	prokka_01835	Hypothetical protein	984
28	prokka_01836	RNA pyrophosphohydrolase	444
29	prokka_01837	Hypothetical protein	519
30	prokka_01838	Hypothetical protein	261
31	prokka_01839	Hypothetical protein	735
32	prokka_01840	Hypothetical protein	738
33	prokka_01841	hypothetical protein	480
34	hns_2	DNA binding protein H_NS	405
35	prokka_01843	Hypothetical protein	549
36	prokka_01844	Hypothetical protein	1,272
37	prokka_01845	Hypothetical protein	297
38	prokka_01846	Hypothetical protein	291
39	prokka_01847	Hypothetical protein	219
40	prokka_01848	Hypothetical protein	876
41	prokka_01849	Hypothetical protein	525

^a^ GenBank accession number CP029486. SGI-1C, Salmonella genomic island 1 type C (bases 427735 to 472096); SPI-23, Salmonella pathogenicity island 23 (bases 2369809 to 2406412).

### Data availability.

This whole-genome assembly sequence was deposited in the NCBI database under the accession number CP029486. The SRA accession numbers are SRX3643218 (Illumina reads) and SRX4523973 (PacBio reads).

## References

[1] European Food Safety Authority. 2017 The European Union summary report on trends and sources of zoonoses, zoonotic agents and food-borne outbreaks in 2016. EFSA J 15:e05077. doi:10.2903/j.efsa.2017.5077. PMC7009962 32625371

[2] Hayward MR, AbuOun M, La Ragione RM, Tchorzewska MA, Cooley WA, Everest DJ, Petrovska L, Jansen VA, Woodward MJ 2014 SPI-23 of *S.* Derby: role in adherence and invasion of porcine tissues. PLoS One 9:e107857. doi:10.1371/journal.pone.0107857. 25238397 PMC4169617

[3] Sévellec Y, Vignaud M-L, Granier SA, Lailler R, Feurer C, Le Hello S, Mistou M-Y, Cadel-Six S 2018 Polyphyletic nature of *Salmonella enterica* serotype Derby and lineage-specific host-association revealed by genome-wide analysis. Front Microbiol 9:891. doi:10.3389/fmicb.2018.00891. 29867804 PMC5966662

[4] Grimont PAD, Weill FX 2007 Antigenic formulae of the *Salmonella* serovars, 9th ed WHO Collaborating Center for Reference and Research on *Salmonella*, Institut Pasteur, Paris, France.

[5] Andrews S 2010 FastQC: a quality control tool for high throughput sequence data. http://www.bioinformatics.babraham.ac.uk/projects/fastqc.

[6] Schmieder R, Edwards R 2011 Quality control and preprocessing of metagenomic datasets. Bioinformatics 27:863–864. doi:10.1093/bioinformatics/btr026. 21278185 PMC3051327

[7] Chin C-S, Alexander DH, Marks P, Klammer AA, Drake J, Heiner C, Clum A, Copeland A, Huddleston J, Eichler EE, Turner SW, Korlach J 2013 Nonhybrid, finished microbial genome assemblies from long-read SMRT sequencing data. Nat Methods 10:563–569. doi:10.1038/nmeth.2474. 23644548

[8] Myers EW, Sutton GG, Delcher AL, Dew IM, Fasulo DP, Flanigan MJ, Kravitz SA, Mobarry CM, Reinert KH, Remington KA, Anson EL, Bolanos RA, Chou HH, Jordan CM, Halpern AL, Lonardi S, Beasley EM, Brandon RC, Chen L, Dunn PJ, Lai Z, Liang Y, Nusskern DR, Zhan M, Zhang Q, Zheng X, Rubin GM, Adams MD, Venter JC 2000 A whole-genome assembly of *Drosophila*. Science 287:2196–2204. doi:10.1126/science.287.5461.2196. 10731133

[9] Li H 2011 A statistical framework for SNP calling, mutation discovery, association mapping and population genetical parameter estimation from sequencing data. Bioinformatics 27:2987–2993. doi:10.1093/bioinformatics/btr509. 21903627 PMC3198575

[10] Seemann T 2014 Prokka: rapid prokaryotic genome annotation. Bioinformatics 30:2068–2069. doi:10.1093/bioinformatics/btu153. 24642063

