# Correction for Sévellec et al., “Complete Genome Sequence of Salmonella enterica subsp. *enterica* Serotype Derby, Associated with the Pork Sector in France”

**DOI:** 10.1128/MRA.01597-18

**Published:** 2019-01-03

**Authors:** Yann Sévellec, Sophie A. Granier, Nicolas Radomski, Arnaud Felten, Simon Le Hello, Carole Feurer, Michel-Yves Mistou, Sabrina Cadel-Six

**Affiliations:** a Université Paris-Est, Marne-la-Vallée, France; b Institut Pasteur, Centre National de Référence des Salmonella, Unité des Bactéries Pathogènes Entériques, Paris, France; c French Institute for the Pig and Pork Industry (IFIP), Le Rheu, France; d ANSES, Laboratory for Food Safety, Maisons-Alfort, France

## AUTHOR'S CORRECTION

Volume 7, no. 12, e01027-18, 2018, https://doi.org/10.1128/MRA.01027-18. Page 1: The author byline should appear as given in this correction.

